# Microdissection and Chromosome Painting of the Alien Chromosome in an Addition Line of Wheat - *Thinopyrum* *intermedium*

**DOI:** 10.1371/journal.pone.0072564

**Published:** 2013-08-14

**Authors:** Chuanliang Deng, Lili Bai, Shulan Fu, Weibo Yin, Yingxin Zhang, Yuhong Chen, Richard R.-C. Wang, Xiangqi Zhang, Fangpu Han, Zanmin Hu

**Affiliations:** 1 Institute of Genetics and Developmental Biology, Chinese Academy of Sciences, Beijing, People’s Republic of China; 2 Henan Normal University, Xinxiang, Henan, People’s Republic of China; 3 Graduate University of Chinese Academy of Sciences, Beijing, People’s Republic of China; 4 Department of Agriculture, ARS, FRRL, Utah State University, Logan, Utah, United States of America; Duke University, United States of America

## Abstract

In this study, chromosome painting was developed and used to identify alien chromosomes in TAi-27, a wheat - *Thinopyrum* *intermedium* addition line, and the chromosomes of the three different genomes of *Th. Intermedium*. The smallest alien chromosome of TAi-27 was microdissected and its DNA amplified by DOP-PCR was used as a probe to hybridize with metaphase chromosomes of TAi-27 and *Th* *. intermedium* . Results showed that hybridization signals were observed in all regions of a pair of the smallest alien chromosomes and the pericentromeric area of another pair of alien chromosomes in TAi-27, indicating that the probe from microdissected chromosome is species specific. In *Th* *. intermedium* , 14 chromosomes had wide and strong hybridization signals distributed mainly on the pericentromere area and 9 chromosomes with narrow and weak signals on the pericentromere area. The remaining chromosomes displayed a very weak or no signal. Sequential FISH/GISH on *Th* *. intermedium* chromosomes using the DNAs of microdissected chromosome, *Pseudoroegneria* *spicata* (**St** genome) and pDbH12 (a **J^s^** genome specific probe) as the probes indicated that the microdissected chromosome belonged to the **St** genome, three genomes (**J^s^**, **J** and **St**) in *Th* *. intermedium* could be distinguished, in which there is no hybridization signal on **J** genome that is similar to the genome of *Th* *. bessarabicum* . Our results showed that the smallest alien chromosomes may represent a truncated chromosome and the repetitive sequence distribution might be similar in different chromosomes within the **St** genome. However, the repetitive sequence distributions are different within the J^s^ genome, within a single chromosome, and among different genomes in *Th* *. intermedium* . Our results suggested that chromosome painting could be feasible in some plants and useful in detecting chromosome variation and repetitive sequence distribution in different genomes of polyploidy plants, which is helpful for understanding the evolution of different genomes in polyploid plants.

## Introduction

The technique of chromosome microdissection and microcloning has become an efficient and direct approach for isolating DNA from specific chromosomes and/or chromosome-specific regions [1], which can be used as the probe for chromosome ‘painting’. Chromosome ‘painting’ refers to the hybridization of fluorescently labelled, chromosome-specific composite probe pools to cytological preparations [2], which has been greatly improved and offers a tool for the identification of chromosomes involved in aneuploidy or rearrangements and location of breakpoint positions of interchromosomal rearrangements [3]. In chromosome painting, probes are most commonly obtained from chromosome flow sorting or micro-manipulated dissection of metaphase chromosomes subjected to PCR amplification using degenerate primers [4,5]. Chromosome painting using a probe obtained from chromosome micro-dissection has been well established in humans and other mammals, and the technique provides an important tool for identifying chromosome origins and aberrations as well as a comparative analysis of genomes [6–10].

Unlike in mammalian systems, chromosome painting in plants is relatively underdeveloped, with only a few successful studies reported. Using the probes amplified from microdissected B chromosomes of *Secale* *cereale* and *Brachycome* *dichromosomatica* , B chromosomes can be distinguished from A chromosomes [11,12]. In other species, sex chromosomes can also be identified using painting probes obtained by chromosome micro-dissection [13,14]. However, with the exception of these examples, microdissected chromosomes cannot be clearly distinguished from other chromosomes using the chromosome painting method because the hybridization signals from the microdissected chromosome and other chromosomes in the same cells are almost the same [3,15,16].

In examining previous reports on plant chromosome painting, we found that the probe from microdissected chromosomes was usually used to hybridize the chromosomes from the same plant species. There is no report indicating whether or not the probe from microdissected chromosomes is species specific. The species specific probe from microdissected chromosomes can be used to hybridize the chromosomes in chromosome addition and translocation lines to find the similarity and divergence of chromosome repetitive sequences between different species at the molecular cytogenetic level. In addition, the research on plant chromosome painting was limited to only a few plant species. Therefore, more species should be further investigated for identifying the sequence distribution patterns on different chromosomes and/or different regions of the same chromosome.

*Thinopyrum* *intermedium* (Host) Barkworth and D.R. Dewey (2*n* = 6*x* = 42) (syn. *Agropyron* *intermedium* (Host) Beauvoir; syn. *Elytrigia* *intermedia* (Host) Nevski) has been used extensively for hybridization with bread wheat and durum wheat. Numerous useful genes, particularly those coding for leaf and stem rust resistance, have been successfully transferred to wheat [17–20]. Two sets of wheat – *Thinopyrum* *intermedium* alien addition lines have been established in China [21]. TAi-27 is one of 14 alien addition lines (2n = 44) carrying at least a pair of chromosomes from *T* *. intermedium* in common wheat [21], which possesses resistance to *barley yellow drarf virus* (BYDV) located on the alien chromosomes [22–24]. Later, TAi-27 was found to possess two pairs of **St** genome chromosomes, one disomic addition pair and another pair substituting for a pair of wheat chromosomes [23,24]. Zhang et al. (2001) showed that a group 2 **St** chromosome derived from the same source (partial amphiploid Zhong 4 awnless) as TAi-27 is responsible for BYDV resistance [25]. Therefore, it appears that the BYDV resistance-bearing chromosome in TAi-27 is the same group 2 chromosome as that in addition line Z1 [26,27]. Based on the above research background, TAi-27 is a useful material for a chromosome painting study using probes generated following microdissection.

In this study, chromosome painting was conducted using the smallest alien chromosome of TAi-27 (microdissected, followed by DOP-PCR) as the probe and the metaphase chromosomes of TAi-27 and *Th* *. intermedium* as the templates. The results showed that 1) the probe is **St** genome- specific in the common wheat background and alien chromosomes, and their structural aberrations can be clearly identified in TAi-27 and 2) the repeat sequences in the probe distributed in a wide pericentromeric area of chromosomes in the **St** genome, in a smaller centromeric area of 9 of 14 **J^s^** chromosomes, and are absent in the third chromosome group (J), suggesting that the distribution of repeat sequence should be region specific in homologous chromosomes and significantly different in different genomes in *Th* *. intermedium* . This study suggested that chromosome painting can be used to identify alien chromosomes, including chromosome number and structural aberration, in wheat hybrids involving alien species. It can also be used to investigate the sequence and distribution differences within homologous chromosomes, between different homologous groups in a polyploid species, or between different plant species.

## Results

### Individual alien chromosome identification, microdissection and amplification

**Figure 1:**
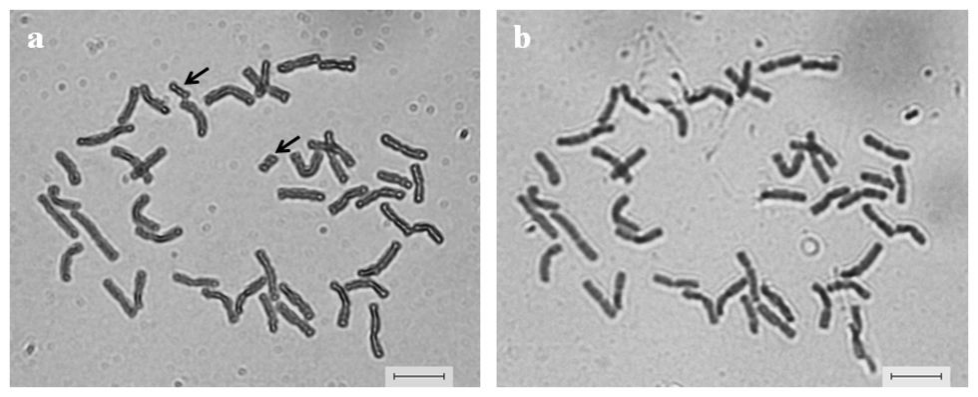
The isolation of alien chromosomes in TAi-27 by a micromanipulator. a Mitosis metaphase chromosomes of a root tip cell before chromosome isolation (Arrows show the smallest chromosome). b Metaphase chromosomes after isolation of the chromosome indicated by arrows in (a) by a glass needle. Bar = 10 µm.

In the metaphase chromosomes of TAi-27, the smallest alien chromosome can be easily identified (Figure 1a). The smallest submetacentric chromosome was successfully microdissected by a fine glass needle (Figure 1b). An isolated single chromosome was separately collected into a tube and used for DOP-PCR. After two successive rounds of amplification, the quality of the obtained PCR product was checked on the agarose gel. Bright smear bands with a range from 250 bp to 2,000 bp were detected (Figure S1, lanes 2 to 5). As a negative control for monitoring possible contamination with DNA, a sample without template DNA was set up during all stages of the microdissection and amplification procedures. No product was amplified from the negative control (Figure S1, lane 1).

### GISH analysis of TAI-27

**Figure 2:**
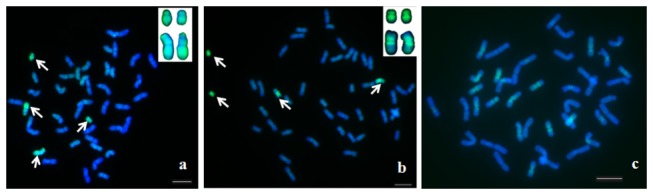
GISH and chromosome painting of mitotic metaphase chromosomes of TAi-27 and *Th* *. intermedium* . a Identification of alien chromosomes in TAi-27 by GISH with the probe, Chroma Tide Alexa Fluor 488-5-dUTP-labeled genomic DNA of *Th*. *Intermedium* (blocked with genomic DNA of “Chinese Spring”). b Identification of alien chromosomes in TAi-27 by chromosome painting with the probe, Chroma Tide Alexa Fluor 488-5-dUTP-labeled DOP-PCR products of the microdissected chromosome in TAi-27. c Chromosome painting of *Th* *. intermedium* chromosomes using Chroma Tide Alexa Fluor 488-5-dUTP-labeled DOP-PCR products of the microdissected chromosome in TAi-27 as the probe. The alien chromosomes in TAi-27 are indicated by arrows and enlarged on the right corner in (a) and (b), respectively. Bar = 10 µm.

Using *Th* *. intermedium* and *Ps* *. spicata* genomic DNA as the probe and “Chinese Spring” genomic DNA as the competitor DNA, the alien chromosomes in TAi-27 were identified (Figure 2a and S2). The results showed that strong hybridization signals were uniformly distributed on two pair of chromosomes, of which one pair of chromosomes were the smallest in TAi-27. This observation indicated that the two pairs of chromosomes from *T* *. intermedium* belonged to the **St** genome. The results confirmed a previous report [23].

### Chromosome painting of alien chromosome

The Chroma Tide Alexa Fluor 488-5-dUTP labelled DOP-PCR product from microdissested chromosome was hybridized to the chromosomes of TAi-27 and *Th* *. intermedium* in the absence of competitor DNA. Signals were observed in all regions of a pair of the smallest alien chromosomes and in the pericentromeric area of another pair of alien chromosomes in TAi-27 (Figure 2b). In *Th* *. intermedium* , the wide and strong fluorescence signals were distributed on the pericentromeric area of 14 chromosomes, tightly restricted and weak on the pericentromeric area of 9 chromosomes and very weak or absent on the remaining chromosomes (Figure 2c).

**Figure 3:**
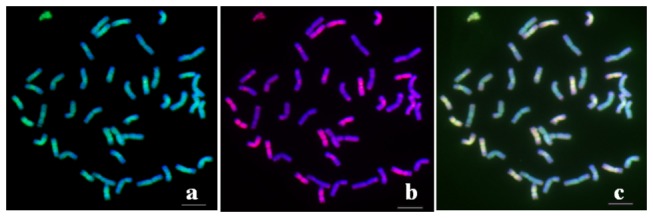
Sequential FISH and GISH on the root-tip cells at mitotic metaphase in *Th* *. intermedium* . a The hybridization results using the DOP-PCR products of the smallest alien chromosome of TAi-27 (green) as the probe. b The hybridization results using genomic DNA from *Ps* *. spicata* (**St** genome, red) as the probe. **C** The merged figure of (a) and (b). Bar = 10 µm.

To determine whether the DNA of the microdissected smallest alien chromosome in TAi-27 is from the **St** genome, a sequential FISH/GISH was conducted using amplified DNA from microdissected alien chromosome and *Ps* *. spicata* genomic DNA (**St** genome) as the probe. The results showed that the hybridization signal distribution pattern of the microdissected smallest alien chromosome DNA was similar to that of genomic DNA from *Ps* *. spicata* (Figure 3), indicating that the microdissected smallest alien chromosomes in TAi-27 are from the **St** genome.

**Figure 4:**
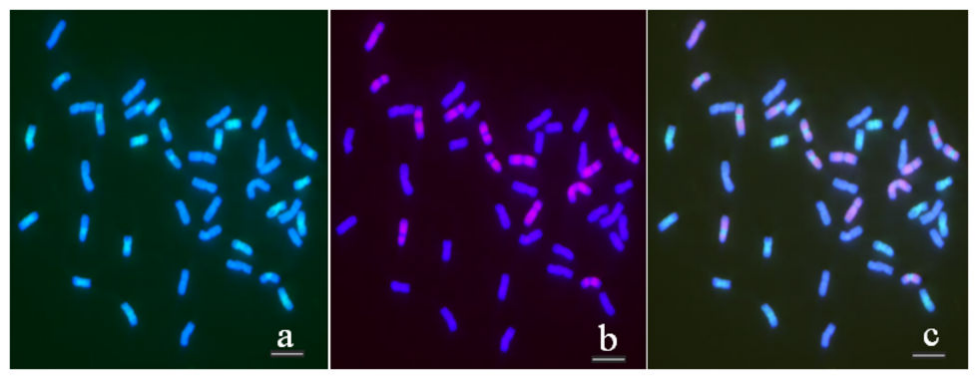
FISH patterns on the root tip cells at mitotic metaphase in *Th* *. intermedium* using (a) the DOP-PCR products of the smallest alien chromosome of TAi-27 (green) and (b) pDbH12 (J^s^ genome specific probe, red) as the probe. **C** The merged figure of (a) and (b). Bar = 10 µm.

To identify the genomic origin of chromosomes with small areas of weak hybridization signals when hybridized with the microdissected chromosome DNA in *Th* *. intermedium* , a sequential FISH was conducted using amplified DNA from the mcirodissected alien chromosome in TAi-27 and pDbH12 (a J^s^ genome specific probe) as the iterative probes. The result indicated that 9 chromosomes with small areas of weak hybridization signal originated from the J^s^ genome (Figure 4).

**Figure 5:**
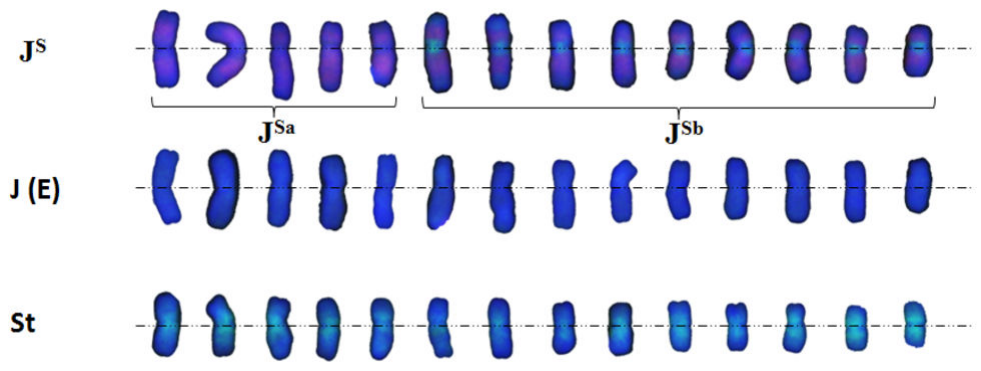
The karyotype of chromosomes of *Th* *. intermedium* from Figure 4c.

We manually organized *Th* *. intermedium* chromosomes (Figure 5) according to Figure 4c. The chromosomes of *Th* *. intermedium* can be divided into three groups. Group 1 included 14 J^s^ chromosomes with strong hybridization signals when using pDbH12 as the probe, of which 9 chromosomes had small areas of weak hybridization signal in the centromeric region when using microdissected alien chromosome DNA as the probe. Group 2 included 14 J (**E**) chromosomes with very weak or absent hybridization signal when using either microdissected chromosome DNA of TAi-27 or pDbH12 as the probe. Group 3 included 14 **St** chromosomes with broadly distributed, strong hybridization signals when using microdissected chromosome DNA of TAi-27 as the probe.

## Discussion

In human and animal samples, chromosome painting with microdissected chromosome as a probe had been successfully used to identify chromosome structural aberrations and origins and to compare the differences between genomes. However, previous chromosome painting studies in plant unequivocally indicated that the hybridization signal dispersed on all chromosomes when using DOP-PCR amplified probes from microdissected chromosomes [3,15,16,28]. Fuchs et al. (1996) concluded that chromosome painting of homologous/homoeologous chromosomes was not yet possible in higher plants with large, complex genomes [3]. Schubert et al. (2001) believed that the failure of plant chromosome painting was due to extensive sequence homogenization between the chromosomes of plant genomes by frequent transposition and/or conversion events [15]. In our study, in which we used DOP-PCR products of the microdissected single smallest chromosome in TAi-27 as probes, strong hybridization signals were distributed on all regions of the smallest pair of alien chromosomes and pericentromere area of another pair of alien chromosomes in TAi-27 (Figure 2b). Wide and strong hybridization signals were distributed on pericentromere area of 14 chromosomes, tightly restricted and weak signal was observed on 9 chromosomes, and a very weak or no signal was detected on the remaining chromosomes in *Th* *. intermedium* (Figure 5). Our study suggested that chromosome painting could be feasible in some plants, such as some progenies from wide crosses and polyploid plants.

**Figure 6:**
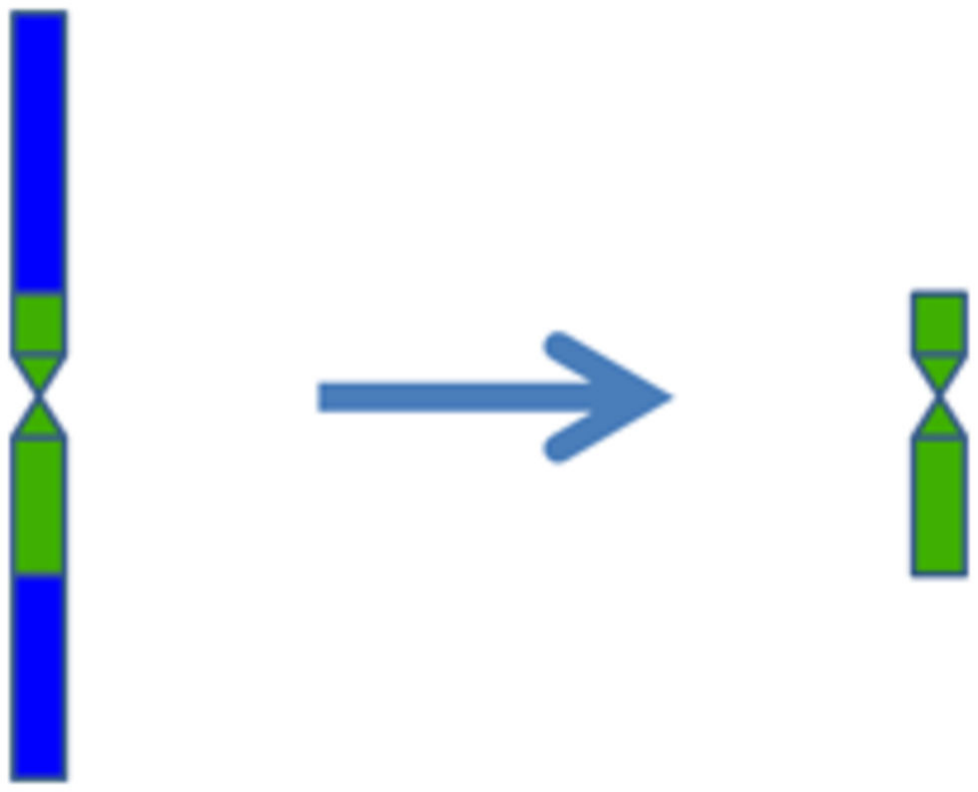
Ideogram showing how the pair of chromosomes from *Th* *. intermedium* changed into the smallest submetacentric chromosome in TAi-27. **St** chromosome breakages occurred, and only the centromeric area remained. A new submetacentric chromosome was subsequently formed.

In plant chromosome painting, the hybridization signal on chromosomes could be indicative of the distribution of repetitive sequences in the probe [3]. The fact that hybridization fluorescent signals were only distributed on the pericentromere area of a pair of alien chromosomes in TAi-27 (Figure 2b) indicated that the repetitive sequences distribution varied in different regions of non-homologous chromosomes in *Th* *. intermedium* . This study showed that chromosome painting could be used to detect alien chromosome structural changes that occurred after the chromosomes of *Th* *. intermedium* were introgressed into wheat. TAi-27 was confirmed to have two pairs of alien chromosomes, one being a disomic addition pair and another substituting for a pair of wheat chromosomes [23–25]. Han et al. (1998) reported that a pair of chromosomes from *Th* *. intermedium* corresponded to small, submetacentric chromosomes in TAi-27 [23]. In this study, we further confirmed previous reports by the GISH technique (Figure 2a). However, the fluorescence signals were distributed on all regions of the smallest chromosomes and another pair of alien chromosomes; the alien chromosome structural change could not be directly determined by GISH. Our chromosome painting results suggested that the chromosome breakage occurred after the alien chromosome was transferred into wheat (Figures 2 and 6). This is the first evidence for the alien chromosome structural change in TAi-27 using chromosome painting. In mammals and humans, chromosome painting technique had been successfully used to identify chromosomal rearrangements [7–9]. However, in plants, there has been no any report on identifying chromosome structural aberrations by using chromosome painting. Chromosome structural aberrations often happened in the process of improving crop by distant hybridization. But, some of chromosome structural aberrations cannot be directly identified by GISH [23]. Our research showed that chromosome painting technique would provide a new way to identify chromosome structural variation in distant hybrid plants.

According to previous reports, the genome of *Th* *. intermedium* consists of two closely related genomes similar to those of *Th* *. elongatum* and *Th* *. bessarabicum* and one distant genome similar to that of *Ps* *. strigosa* [29–33]. Therefore, the 42 chromosomes of *Th* *. intermedium* are composed of three genomes. Our sequential FISH and GISH on the metaphase chromosomes of *Th* *. intermedium* showed that the fluorescence signal distribution patterns of the probe made from microdissected chromosome DNA of TAi-27 and that made from **St** genomic DNA were similar (Figure 3). The probe made from microdissected chromosome DNA of TAi-27 can be hybridized with most of the J^s^ chromosomes (Figure 4). Our observations suggested that the **St** genome is more closely related to the J^s^ genome than to the **J** (**E**) genome in *Th* *. intermedium* . The results indicated that chromosome painting can be used to identify the repetitive sequence distribution on chromosomes from different genomes in *Th* *. intermedium* .

Furthermore, our results showed that the 42 chromosomes of *Th* *. intermedium* can be divided into three groups (Figure 5). In group 1, 9 chromosomes (and not the other 5) had tightly restricted and weak hybridization signals in the centromeric region when using microdissected chromosome DNA of TAi-27 as the probe, indicating that the repetitive sequences distribution varied in the different chromosomes of J^s^. We named these 9 chromosomes as sub-group **J^sa^** and the left 5 chromosomes of J^s^ as sub-group J^sb^. Although this **J^s^** genome could be hybridized by **V** genomic DNA ( *Dasypyrum* *villosum* ) [33,34] or **V** genome-derived pDbH12 (Figure 6b of this study), **J^s^** could not be the present-day **V** genome because of differences in chromosome size and karyotype – the **V**-genome chromosomes are much shorter than those of the **J^s^** genome. The **J^s^** genome is more likely a progenitor of the **J/E** genome with introgressed **St** genome centromeric sequences. In group 2, the 14 chromosomes barely hybridized with the microdissected chromosome DNA, indicating that the **J** (**E**) genome could be discriminated from the J^s^ and **St** genomes by FISH. In group 3, the 14 chromosomes strongly hybridized with the microdissected chromosome DNA of TAi-27 as well as genomic DNA of the **St** genome. The third genome in *Th* *. intermedium* is unequivocally the **St** genome [29–33].

In conclusion, plant chromosome painting can be used to detect alien chromosome variation in wheat distant hybrids. We found that the repetitive sequence distribution may be 1) similar in different chromosomes within the **St** genome, but different within the J^s^ genome, 2) different within a single chromosome, and 3) different among the different genomes in *Th* *. intermedium* . Our findings are helpful in furthering our understanding of the evolution of different genomes in polyploid plants, such as *Th* *. intermedium* of Triticeae.

## Materials and Methods

Plants used in this research included wheat *Thinopyrum* alien addition line TAi-27(2n = 44), *Th* *. intermedium* (**J^s^J^s^J(E) J(E) StSt**, 2n = 42), and *Pseudoroegneria* *spicata* (2n = 14, **StSt**).

### Chromosome microdissection and DNA amplification of the alien chromosome of TAi-27

The chromosome microdissection of the alien chromosome in TAi-27 was performed according to the procedures described by Jiang et al. (2009) [35]. In brief, the alien chromosomes in TAi-27 were identified by their size (Figure 1a) and then microdissected using a glass needle fixed on the arm of a Leitz micro-operation instrument on an inverted phase contrast microscope (Olympus 1M, Japan). The microdissected chromosome was collected into a 0.2 ml Eppendorf tube and digested with 10 µL (0.5 mg/ml) of proteinase K (Roche, Indianapolis) at 37° C for 2 h in 1 × *Taq* Polymerase buffer (Promega, Madison). The proteinase K was then inactivated at 90° C for 10 min.

The chromosome DNA was amplified by DOP-PCR in a PTC-200 thermocycler (MJ Research, Watertown, MA, USA). The DOP primer sequence was 5’-CCG ACT CGA GNN NNN NAT GTG G-3’. Two rounds of PCR were performed. The first round of PCR was conducted in the original tube by adding 5 µL of 10× *Taq* Polymerase buffer (Promega, Madison), 3 µL of 25 mM MgCl_2_ (Promega, Madison), 2.5 U *Taq* DNA Polymerase (Promega, Madison), 1 µL of 10 mM dNTPs (MBI, Lithuania), 1 µL of DOP primer (10 µm) and double-distilled water to a total volume of 50 µL. Denaturation was performed at 94°C for 10 min, followed by 5 low-stringency cycles of 94°C for 1 min, 30°C for 1.5 min, and 72°C for 3 min; then 25 high-stringency cycles of 94°C for 1 min, 55°C for 1 min, and 72°C for 1.5min; and a final extension at 72°C for 10 min. The second round of PCR was conducted under the same conditions described above, except that only a 5 µL product from the first round of PCR was used as the template and the 5 low-stringency cycles were not performed. In all the procedures, strict negative control experiments were conducted using the same conditions without the templates (Figure S1). PCR products were analysed in 1% agarose gels.

### Genomic in situ hybridization (GISH)

Seeds of TAi-27 and *Th* *. intermedium* were germinated on moistened filter paper in petri dishes. Actively growing roots were removed from seedlings and treated by N_2_O for 2 h, fixed in 90% acetic acid and stored in 70% v/v ethanol. The chromosome spread preparation was performed as previously described [36]. Genomic DNA of *Th* *. intermedium* and *Ps* *. spicata* was isolated by a modified CTAB method [37] and labelled with Chroma Tide Alexa Fluor 488-5-dUTP and Texas Red-5-dCTP respectively by the nick translation method and subsequently used as a probe. Detection and visualization were performed as described by Han et al. (2009) [38].

### Fluorescence in situ hybridization (FISH)

The metaphase chromosomes of TAi-27 and *Th* *. intermedium* were prepared using the method described by Han et al. (2006) [36]. The second-round PCR products from microdissected single chromosome preparations were labelled with Chroma Tide Alexa Fluor 488-5-dUTP by the nick translation method and used as a probe. A new *Sabrina*-like long terminal repeat (LTR) pDbH12 isolated from *Dasypyrum* *breviaristatum* [39] was labelled with Texas Red-5-dCTP and used as a cytogenetic marker for tracing the J^s^ genome in *Th* *. intermedium* . The probe solution (20 ng/µL in 2 × SSC and 1 × TE buffer) was denatured for 5 min in boiling water and then placed on ice for 10 min. Slides were UV cross-linked for 2 min (a total energy of 120–125 mJ per square cm was delivered). After total 6 µL of probe solution was spread onto each slide and covered with a plastic coverslip, the slides were heated for 5 min at 100°C and then incubated at 55°C overnight in a humid chamber. After hybridization, the slides were washed using 2 × SSC for 5 min and then mounted with Vectashield mounting medium (containing 1.5 µg/mL DAPI, Vector Laboratories, Burlingame, CA, USA). The FISH images were captured with a Magnafire CCD camera under a Zeiss Universal microscope (Zeiss, Jena, Germany) and processed with Photoshop 7.0.

## Supporting Information

Figure S1 **DOP-PCR products using a microdissected alien chromosome of TAi-27 as the template.** M The DNA molecular weight marker. Lane 1 Negative control. Lane 2, 3, 4 and 5 PCR product using the single microdissected alien chromosome as the template.
(TIF)

Figure S2 **GISH on a root** **tip cell at mitotic metaphase in TAi-27**. The alien chromosomes were detected by GISH with Texas Red-5-dCTP labelled genomic DNA of *Ps* *. spicata* (**St** genome, red) (blocked with genomic DNA of “Chinese Spring”). The alien chromosomes from **St** genome are indicated by arrows and enlarged on the right corner. Bar = 10 µm.
(TIF)
