# Supplementary figures and images for: Microdissection and Chromosome Painting of the Alien Chromosome in an Addition Line of Wheat - Thinopyrum intermedium
Source: PLoS One. 2013 Aug 14;8(8):e72564. doi: 10.1371/journal.pone.0072564 (PMC3743814; doi:10.1371/journal.pone.0072564)

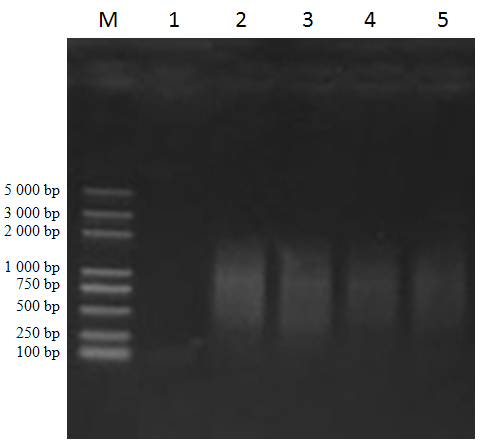

Supplement: Figure S1 — DOP-PCR products using a microdissected alien chromosome of TAi-27 as the template. M The DNA molecular weight marker. Lane 1 Negative control. Lane 2, 3, 4 and 5 PCR product using the single microdissected alien chromosome as the template. (TIF) [file pone.0072564.s001.tif]

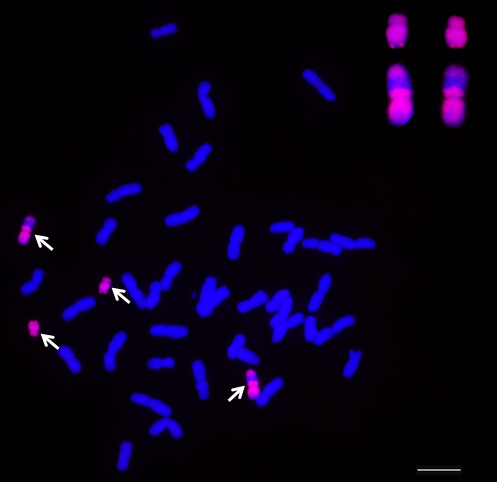

Supplement: Figure S2 — GISH on a root tip cell at mitotic metaphase in TAi-27. The alien chromosomes were detected by GISH with Texas Red-5-dCTP labelled genomic DNA of Ps . spicata (St genome, red) (blocked with genomic DNA of “Chinese Spring”). The alien chromosomes from St genome are indicated by arrows and enlarged on the right corner. Bar = 10 µm. (TIF) [file pone.0072564.s002.tif]
